# Investigating heart failure mechanics using personalised human biophysical models

**DOI:** 10.1186/1532-429X-15-S1-E32

**Published:** 2013-01-30

**Authors:** Vicky Y Wang, Brett R Cowan, Alistair A Young, Martyn P Nash

**Affiliations:** 1 Auckland Bioengineering Institute, University of Auckland, Auckland, New Zealand; 2 Center for Advanced MRI, University of Auckland, Auckland, New Zealand; 3 Department of Engineering Science, University of Auckland, Auckland, New Zealand

## Background

Heart failure (HF) is a pathological condition during which the pump function of the heart is compromised. There are two major forms: diastolic and systolic HF. Whilst the majority of patients exhibit systolic HF (reduced ejection fraction), diastolic HF (normal ejection fraction) has also been found in almost half of HF patients. A major question in this field is the inter-relationship between diastolic and systolic HF. The mechanisms underlying both pathologies are still poorly understood.

## Methods

In this study, personalised biomechanical models of the human left ventricle (LV) were generated semi-automatically from cardiac magnetic resonance images (MRI) using finite element modelling techniques to investigate both diastolic and systolic ventricular mechanics. Geometric information of the LV throughout the cardiac cycle was derived via semi-automatic segmentation technique called guide-point modelling with myocardial volume preserved throughout the cardiac cycle. After image segmentation, the guide-point model at diastasis was used as the reference mechanics model to simulate ventricular mechanics. Passive and contractile myocardial mechanical properties were then tuned to best match the segmented surface data at end-diastole and end-systole, respectively. Global and regional indices of myocardial mechanics, including muscle fibre stress and extension ratio were then quantified and analysed.

## Results

This mechanics modelling framework was applied to the Sunnybrook Cardiac Database which consisted of normal, hypertrophy and non-ischemic HF cases. Comparison of the estimated passive stiffness and peak active contractile stress between the normal and diseased cases provided some preliminary insight into the changes in myocardial mechanical properties during heart failure. It was found that the passive tissue stiffness was greater for the HF and hypertrophy patients compared to the normal subjects. This is consistent with fibrosis that is observed during failure. On the other hand, the estimated maximum active contractile stress did not vary significantly between the diseased and healthy cases which may suggest that systolic heart failure may not be associated with changes in systolic mechanical properties.

## Conclusions

This automated approach enables minimally invasive personalised characterisation of cardiac mechanical function in health and disease. Determining more specific variations in mechanical properties between diseased and normal human hearts will help in the understanding of the underlying mechanisms of HF.

## Funding

This work is funded by the Health Research Council of New Zealand.

